# Postoperative Trapped Lung After Orthotopic Liver Transplantation is a Predictor of Increased Mortality

**DOI:** 10.3389/ti.2022.10387

**Published:** 2022-05-03

**Authors:** Natasha Cuk, Kathryn H. Melamed, Sitaram Vangala, Ramy Salah, W. Dwight Miller, Sarah Swanson, David Dai, Zarah Antongiorgi, Tisha Wang, Vatche G. Agopian, Joseph Dinorcia, Douglas G. Farmer, Jane Yanagawa, Fady M. Kaldas, Igor Barjaktarevic

**Affiliations:** ^1^ Department of Medicine, David Geffen School of Medicine, University of California, Los Angeles, Los Angeles, CA, United States; ^2^ Division of Pulmonary and Critical Care Medicine, Department of Medicine, David Geffen School of Medicine, University of California, Los Angeles, Los Angeles, CA, United States; ^3^ Clinical and Translational Science Institute, David Geffen School of Medicine, University of California, Los Angeles, Los Angeles, CA, United States; ^4^ Division of Critical Care Medicine, Department of Anesthesiology, David Geffen School of Medicine, University of California, Los Angeles, Los Angeles, CA, United States; ^5^ Division of Transplant Surgery, Department of Surgery, David Geffen School of Medicine, University of California, Los Angeles, Los Angeles, CA, United States; ^6^ Division of Thoracic Surgery, Department of Surgery, David Geffen School of Medicine, University of California, Los Angeles, Los Angeles, CA, United States

**Keywords:** trapped lung, hepatic hydrothorax, liver transplantation, pleural effusions, pneumothorax-ex-vacuo

## Abstract

Pleural effusions are a common complication of orthotopic liver transplantation (OLT), and chronic post-OLT pleural effusions have been associated with worse outcomes. Furthermore, “trapped lung” (TL), defined as a restrictive fibrous visceral pleural peel preventing lung re-expansion, may have prognostic significance. We performed a retrospective analysis of adult OLT recipients over a 9-year period at UCLA Medical Center. Post-OLT patients with persistent pleural effusions, defined by the presence of pleural fluid requiring drainage one to 12 months after OLT, were included for analysis. Outcomes for patients with and without TL were compared using univariate and multivariate analysis. Of the 1722 patients who underwent OLT, 117 (7%) patients met our criteria for persistent postoperative pleural effusion, and the incidence of TL was 21.4% (25/117). Compared to patients without TL, those with TL required more surgical pleural procedures (OR 59.8, 95%CI 19.7–181.4, *p* < 0.001), spent more days in the hospital (IRR 1.56, 95%CI 1.09–2.23, *p* = 0.015), and had a higher risk of mortality (HR 2.47, 95%CI 1.59–3.82, *p* < 0.001) following transplant. In sum, we found that post-OLT TL was associated with higher morbidity, mortality, and healthcare utilization. Future prospective investigation is warranted to further clarify the risk factors for developing postoperative pleural effusions and TL.

## Introduction

Orthotopic liver transplantation (OLT) is the only definitive treatment for end-stage liver disease (ESLD), but even with rigorous patient and donor selection criteria, most patients experience at least one complication post-transplant (1). With improvements in the understanding of pre-transplant risk factors and post-transplant clinical course, 1-year survival rates have steadily improved for liver transplant recipients over the last 3 decades (2, 3).

Pulmonary complications after OLT remain common and are associated with increased morbidity and mortality (4–8). Postoperative pleural effusions are among the most frequently recognized pulmonary complications of OLT, occurring in 39–95% of all patients (4, 6, 9–10). Retrospective chart reviews reveal that most of these pleural effusions are clinically insignificant and resolve quickly after OLT. However, up to 25% persist after transplantation, often in patients with complicated postoperative courses (7–12). None of these small-scale studies, however, have specifically described the etiology, pathogenesis, and clinical impact of persistent post-OLT pleural effusions or identified risk factors for poor outcomes in this population.

Trapped lung (TL) is a complication of persistent pleural effusion defined by chronically atelectatic lung that is unable to expand due to the development of a fibrous visceral pleural peel (10). The diagnosis of TL is made based on radiographic and clinical criteria once drainage of the pleural fluid has been attempted and demonstrates pneumothorax ex-vacuo and thickened visceral pleura on imaging. Manometry, if performed, confirms a sharp decline in pleural pressure with minimal fluid drainage. Exudative effusions resulting from inflammatory and infectious conditions have been identified as a risk factor for the development of fibrinous change in the visceral pleura (13–14). Trapped lung is less commonly associated with ESLD, although a few small studies have shown it to be a complication of hepatic hydrothorax in a small subset of patients (15–17). When it does occur, trapped lung and pleural disease have been shown to be indicative of poor outcomes in liver transplant recipients (15, 18).

To our knowledge, this is the largest study to date to describe the demographic, clinical, biochemical, and radiological characteristics of patients with persistent pleural effusions after OLT. Furthermore, given existing data to suggest an association with adverse outcomes in patients with pre-transplant trapped lung, we also sought to characterize and evaluate clinical outcomes with postoperative trapped lung.

## Patients and Methods

### Study Population

We performed a retrospective chart review of the 1722 patients who received an OLT between January 2006 and October 2015 at Ronald Reagan UCLA Medical Center, a high-volume quaternary liver transplant center performing over 150 transplants annually. Patients were included if they were 18 years or older at the time of transplantation and had a pleural effusion that was present between one and 12 months postoperatively. To be considered clinically relevant, only effusions assessed by invasive approach such as thoracentesis or surgical intervention qualified for inclusion in the study. Two subgroups in this cohort were defined as those with trapped lung (TL) present and those with only persistent pleural effusions in the first year post-OLT. Data collection was approved by the institution’s internal review board (IRB #14-000365) and was performed in accordance with the 2000 Declaration of Helsinki and the Declaration of Istanbul 2008.

### Data Collection

Patient data were entered into a secure database. Patient demographics, including age, sex, transplant Model for End-Stage Liver Disease (MELD) score, and clinical characteristics were recorded. Pleural fluid analysis including cell count and differential, microbiology data, lactate dehydrogenase (LDH) and protein concentration, was collected for the most proximate pleural fluid sampling both before (if available) and after transplantation. Corresponding proximate serum LDH and protein were recorded as well. A transudative effusion was defined by Light’s criteria, which required that the three following criteria were met: serum to pleural protein ratio less than 0.5, serum to pleural LDH ratio less than 0.6, and pleural LDH less than two thirds of the upper limit of the normal serum LDH assay (19). Effusions were classified as exudative if they failed to meet one or more of the prespecified pleural fluid criteria.

All chest imaging was reviewed. Patients’ pre- and post-transplantation pleural effusions were described based on chest imaging. TL was defined by radiological evidence of thickened pleural rind and lack of expansion after drainage (resulting in a pneumothorax ex-vacuo or hydropneumothorax). Additional clinical data, such as a pulmonologist’s clinical documentation and assessment, was also reviewed to ensure concordance with imaging findings.

Both preoperative and postoperative pleural interventions, including thoracentesis, chest tube thoracostomy, and video-assisted thoracoscopic surgery (VATS) decortication were also recorded. Ventilator and hemodialysis dependence pre-OLT were defined as requiring mechanical ventilation or dialysis immediately preceding transplant. Ventilator and hemodialysis dependence post-OLT were defined as requiring these interventions for longer than 2 weeks post-OLT.

In addition to descriptive data of the entire cohort, two primary clinical outcomes were identified: mortality and total number of hospital days in the first year following transplantation. Secondary outcomes included mechanical ventilation for a duration of greater than 2 weeks post-transplantation and the need for multiple pleural procedures including thoracentesis, chest tube placement or VATS decortication and/or pleurodesis.

### Statistical Analysis

Descriptive statistics were reported for the full study sample. Comparisons between TL and non-TL pleural effusion cases were performed using two-sample t-tests for age and transplant MELD, and using chi-squared or Fisher’s exact tests as appropriate for the other qualitative characteristics. Analysis of study endpoints was performed using a propensity score approach, based on inverse probability of treatment weighting (IPTW). Age, gender and transplant MELD were pre-specified for inclusion in the propensity score model. Other variables were selected based on a significance threshold of 0.05, having no more than 10 observations missing, and having event rates of at least 5% in both the TL and non-TL cohorts. Propensity scores were estimated using a logistic regression model. After weighting by propensity scores, the cohorts were again compared using the two-sample tests described above.

Analysis of the mortality endpoint was performed using a Cox proportional hazards model, while analysis of number of days hospitalized in the year following transplant was performed using a negative binomial regression model. All other outcomes were analyzed using logistic regression models with Firth’s penalized likelihood method. The primary model term was trapped lung (yes versus no). Each analysis was performed once using the unweighted cohort and once using the weighted cohort. Variables which remained or became significant after IPTW, and which had sufficient non-missing observations and event rates, were included as controls in the weighted versions of the models. The two-stage approach was chosen to most accurately model the effect of TL on outcomes and adjust for pre-treatment differences between TL and non-TL pleural effusion patients in the setting of a potentially large number of confounders and small number of events, which would avoid overfitting the regression models.

Comparisons between TL and non-TL pleural effusion patients were reported in terms of hazard ratios (HR), incidence rate ratios (IRR), and odds ratios (OR) respectively, along with 95% confidence intervals. p-values less than 0.05 were considered statistically significant. A Kaplan-Meier diagram for the mortality endpoint was produced using R v. 3.6.2 (http://www.r-project.org/). All other analyses were performed using SAS v. 9.4 (SAS Institute Inc., Cary, NC).

## Results

### Characteristics of the Study Cohort

Of the 1722 patients who received a transplant during the study period, a total of 117 (7%) adult patients had a persistent pleural effusion requiring invasive management within the first year after OLT. Baseline characteristics of the study cohort are shown in the first column of Table 1. The mean age was 56 ± 9.4 years and a minority of the patients were female (46/117, 39%). Medical co-morbidities were common, including diabetes mellitus (56/114, 49%) and hepatopulmonary syndrome (16/115, 14%). Diverse etiologies of liver disease were represented, including alcoholic cirrhosis, hepatitis B, hepatitis C, and hepatocellular carcinoma. Transplant MELD scores indicated severe liver disease with a mean score of 34 ± 7.8. Effusions were often present by radiographic criteria prior to transplantation (74/117, 63%). Unfortunately, pleural fluid sampling was only performed in 26 of the patients, making analysis of the preoperative pleural fluid limited. Within this limitation, spontaneous bacterial peritonitis was also common pre-operatively (31/105, 30%), although only 9/105 (9%) had documented positive ascites fluid culture and data was not available for 12 patients. A total of 57/117 (49%) patients were dialysis dependent and 35/117 (30%) were ventilator dependent prior to transplant, while 43/117 (37%) were admitted from home. Postoperatively, the majority of effusions were exudative (90/100, 90%) and need for hemodialysis was common (53/116, 46%).

**TABLE 1:** Baseline characteristics of the study cohort.

	Original Cohort Total	Propensity Score Weighted Cohort		
	(n = 117)	Trapped lung	No trapped lung	*p*-value
Demographics				
**Age, mean ± SD (years)**	56 ± 9.4	56 ± 14	56 ± 9	0.742
**Female**	46/117 (39%)	31%	37%	0.381
Comorbidities				
Diabetes Mellitus	56/114 (49%)	49%	46%	0.697
Hepatopulmonary Syndrome	16/115 (14%)	17%	12%	0.280
Portopulmonary Hypertension^b^	3/115 (3%)	5%	2%	0.298
Etiology of Liver Disease				
Alcoholic Cirrhosis	34/116 (29%)	17%	34%	**0.005**
HBV Cirrhosis	14/115 (12%)	19%	12%	0.116
HCV Cirrhosis	56/117 (48%)	56%	50%	0.381
HCC	43/114 (38%)	53%	38%	**0.023**
Preoperative Characteristics				
**Transplant MELD, mean ± SD**	34 ± 7.8	33 ± 15	34 ± 9	0.573
**Pre-OLT Effusion Present**	74/117 (63%)	69%	64%	0.481
Pre-OLT Exudate^a^	5/16 (31%)	43%	38%	0.760
Pre-OLT Empyema^a^	8/34 (24%)	24%	28%	0.677
**Pre-OLT Thora**	26/112 (23%)	25%	24%	0.835
Pre-OLT Chest Tube^b^	10/111 (9%)	20%	3%	**<0.001**
Pre-OLT Trapped Lung^b^	5/117 (4%)	10%	0%	**<0.001**
Pre-OLT HD Dependence	57/117 (49%)	46%	45%	0.836
Pre-OLT Vent Dependence	35/117 (30%)	36%	28%	0.253
Admitted from Home	43/117 (37%)	39%	39%	0.985
Intraop Chest Tube Placement^b^	5/116 (4%)	16%	0%	**<0.001**
Postoperative Characteristics				
Post-OLT Exudate^a^	90/100 (90%)	95%	86%	**0.031**
Post-OLT Empyema^a^	10/103 (10%)	24%	3%	**<0.001**
Post-OLT HD Dependence	53/116 (46%)	42%	46%	0.565

Characteristics are compared by subgroup (trapped lung vs. no trapped lung) and shown after inverse probability of treatment weighting. Covariates chosen to create the propensity score model are shown in bold. Variables which could not be included in the model due to data constraints are indicated with superscripts. *p*-values reaching significance are also bolded.

HBV, hepatitis B virus; HCC, hepatocellular carcinoma; HCV, hepatitis C virus; HD, hemodialysis; intraop = intraoperative; MELD, Model for End-Stage Liver Disease; OLT, orthotopic liver transplantation; thora = thoracentesis; vent = ventilator.

a More than 10 observations missing.

b Less than 5% event rate observed.

### Trapped Lung Cohort Characteristics and Outcomes

The incidence of TL in those with persistent pleural effusion after OLT was 21.4% (25/117). Five (4%) of these patients had evidence of trapped lung prior to transplant. Data are stratified by the presence of TL after OLT and shown after IPTW in Table 1 (data before and after IPTW are presented in the Supplementary Table S1). Covariates chosen for the model include mean age, gender, transplant MELD, presence of an effusion pre-OLT, and preoperative thoracentesis. Mean age, gender, and transplant MELD were balanced in the original cohort and did not differ significantly after IPTW. In comparison to those without TL, patients with TL were more likely to have preoperative effusions (88% vs. 57%, *p* = 0.008) and more likely to have undergone preoperative thoracentesis (54% vs. 15%, *p* < 0.001), although these findings did not retain significance after IPTW (Supplementary Table S1)*.*

Other clinical characteristics that were not included in the IPTW model are also stratified by the presence of TL (Table 1). None of these differences attained significance after IPTW. Compared to patients without postoperative TL, patients with postoperative TL were more likely to have had preoperative chest tube placement (35% vs. 2%, *p* < 0.001) and carry a preoperative diagnosis of trapped lung (20% vs. 0%, *p* < 0.001) (Supplementary Table S1)*.* Additionally, infectious complications were common, and 56/117 (48%) of all patients had radiographic concern for pneumonia post-OLT, although there was no significant difference between those with and without TL (56% vs. 46%, *p* = 0.489). Among those with radiographic concern for pneumonia, only 21/117 (18%) had clinical suspicion of pneumonia, and again there was no difference between TL and non-TL cohorts (16% vs. 18.5%, *p* = 1.00). Lastly, post-OLT sepsis was present in 30/117 (26%) and not significantly different between groups (32% vs. 24%, *p* = 0.574).

Covariates which became unbalanced after IPTW include the presence of alcoholic cirrhosis, hepatocellular carcinoma, and post-OLT exudative effusion, and these were included in regression analyses as covariates. Covariates which were significantly different before and after IPTW and could not be included in the regression analysis due to missing observations and/or low event rates included presence of chest tube or thoracic surgical intervention pre-OLT, pre-OLT trapped lung, and post-OLT empyema.

### Postoperative Clinical Outcomes

**FIGURE 1:**
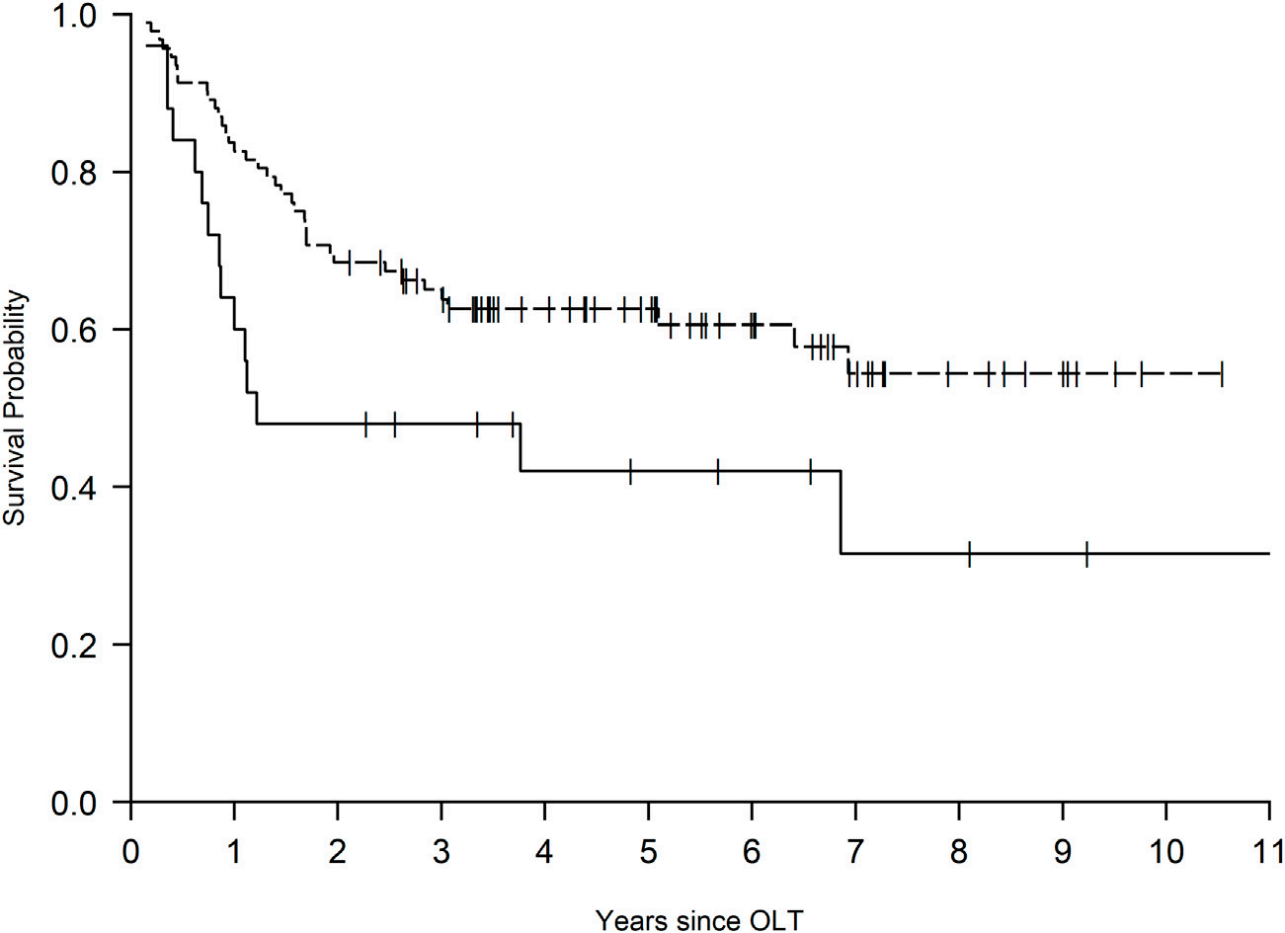
Survival probability of patients with persistent pleural effusion after orthotopic liver transplantation with versus without trapped lung. Kaplan-Meier survival curves demonstrating survival probability based on the presence or absence of postoperative trapped lung. Patients with trapped lung (solid line) have decreased probability of survival compared to patients with chronic postoperative effusion alone (dashed line) (HR 2.47, 95%CI 1.59–3.82, *p* < 0.001).

**FIGURE 2:**
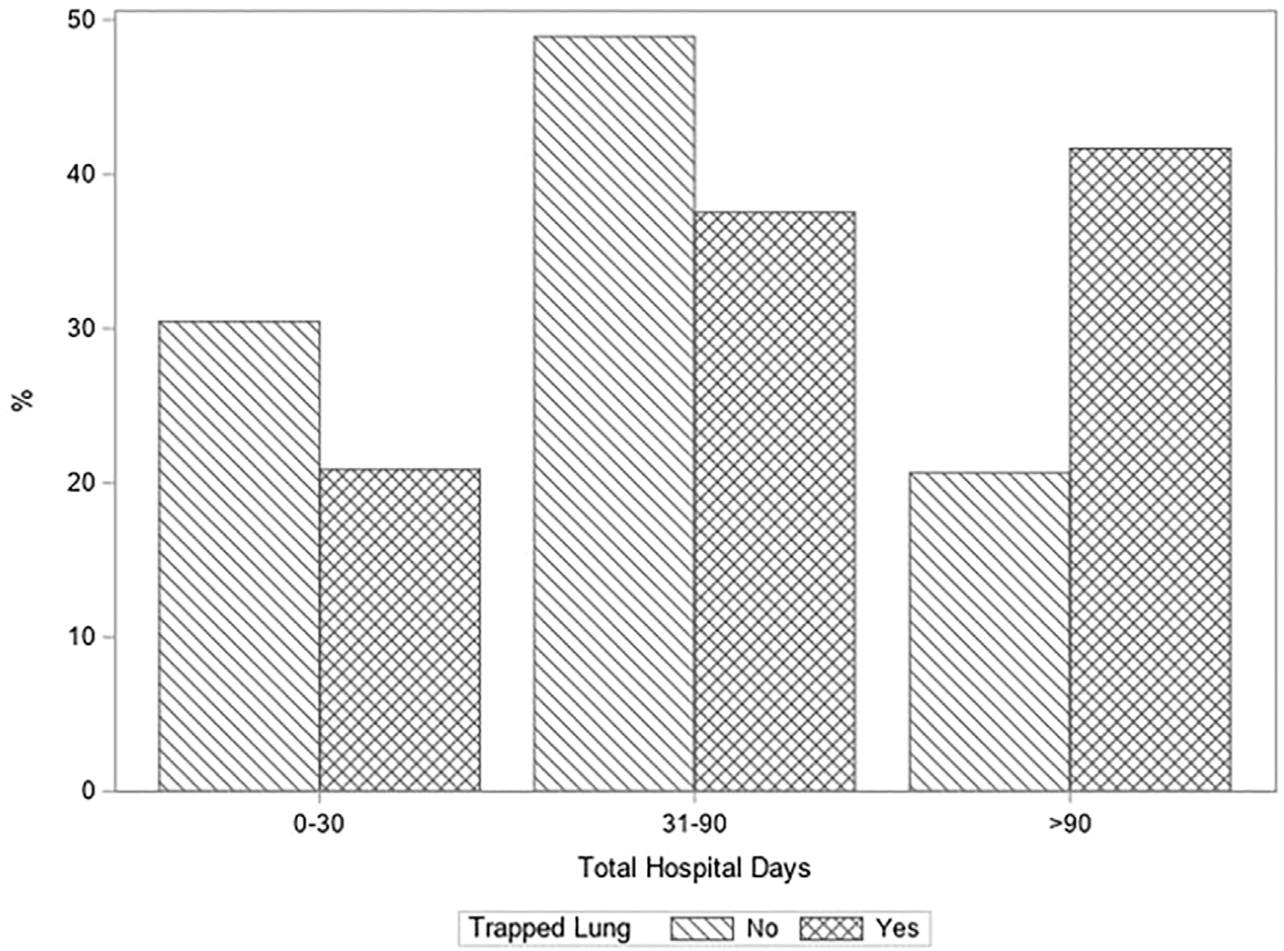
Postoperative total hospital days in the first year for patients with persistent pleural effusion after orthotopic liver transplantation comparing those with versus without trapped lung. Patients with trapped lung spent more days in the hospital in the first year post transplant than those with chronic effusions alone (IRR 1.56, 95%CI 1.09–2.23, *p* = 0.015).

The overall 1-year survival for patients with persistent pleural effusion was 78% (91/117), and compared to simple pleural effusion, those with TL had a significantly higher risk of mortality (HR 2.47, 95%CI 1.59–3.82, *p* < 0.001) (Table 2 and Figure 1). The number of hospital days in the first year post-transplant was also significantly higher for the TL group (IRR 1.56, 95%CI 1.09–2.23, *p* = 0.015). Figure 2 displays these differences in hospitalized days between the two groups as a histogram. These differences were significant both before and after adjustment for differences in baseline characteristics between the groups (Supplementary Table S2).

**TABLE 2:** Clinical outcomes of the study cohort.

	Cohort after IPTW	
	Estimate (95% CI)	*p*-value
Mortality (HR)	2.47 (1.59, 3.82)	**<0.001**
Number of Hospitalized Days in 1st Year Post-Transplant (IRR)	1.56 (1.09, 2.23)	**0.015**
Thoracic Surgical Interventions (OR)	59.8 (19.7, 181.4)	**<0.001**
Multiple Pleural Procedures Post-OLT (OR)	26.8 (6.7, 107.6)	**<0.001**
Ventilator Dependence >2 Weeks (OR)	1.01 (0.54, 1.89)	0.966

Clinical outcomes of the study cohort stratified by the presence of trapped lung after orthotopic liver transplantation and shown after inverse probability of treatment weighting. *p*-values reaching significance are bolded.

HR, hazard ratio; IRR, incident rate ratio; OR, odds ratio.

The TL group also experienced more thoracic surgical interventions (OR 59.8, 95%CI 19.7–181.4, *p* < 0.001) and a higher rate of pleural procedures overall post-OLT (OR 26.8, 95%CI 6.7–107.6, *p* < 0.001). Both of these outcomes were significant before and after adjustment for baseline characteristics as shown in Table 2 and Supplementary Table S2. Ventilator dependence did not differ between the two groups (OR 1.01, 95%CI 0.54–1.89, *p* = 0.966).

## Discussion

Pulmonary complications following OLT are frequent and are associated with increased morbidity and mortality (4–6, 8, 11). While preoperative and early postoperative pleural effusions represent well-described and frequent complications of end-stage liver disease, published evidence about the clinical relevance and outcomes in patients with persistent pleural effusions after an OLT is scant (7,8, 12, 16, 17, 20). In this retrospective analysis, we investigated the clinical relevance of persistent pleural effusions with a specific focus on the risk factors for and complications related to TL, which we found is a common complication of persistent postoperative pleural effusions.

Prior work suggests that most pleural effusions following liver transplantation resolve within 1–3 months (8, 20), but the significance of the remaining persistent effusions is less clear. Further, effusions complicated by TL may portend a different prognosis in comparison to those without TL. For example, in a report of Shirali et al., pleural complications represented the major indication for post-OLT thoracic surgery interventions and were associated with poor outcomes. In the same report, the majority of patients who required thoracic intervention had trapped lung, which was found to be a significant predictor of overall mortality with a 1-year survival of less than 40% (18). In patients with persistent pleural effusion after OLT who undergo diagnostic thoracentesis, the presence of trapped lung may lead to a diagnosis of post-procedural pneumothorax ex-vacuo. This radiographic finding is often interpreted as a procedural complication caused by direct lung injury, rather than an intrinsic lack of lung re-expansion due to a pleural rind. This misinterpretation can lead to further pleural interventions, including tube thoracostomy and surgical intervention, which themselves carry additional risk (17).

In our cohort, we showed that compared to effusions without TL, the presence of TL postoperatively was associated with increased mortality and morbidity, as evidenced by total hospital days, surgical intervention, and pleural procedures within the first postoperative year. To understand why these patients had worse outcomes, we first examined the risk factors for development of trapped lung.

We found that patients with persistent effusions were often diabetic, dialysis-dependent, and had pleural effusion prior to transplant, and qualitatively it appears that spontaneous bacterial peritonitis was also common, together suggesting that there may be some level of chronic systemic illness or inflammatory process not captured by the MELD score that may increase the risk for postoperative effusion. Moreover, trapped lung has been typically associated with inflammatory pleural conditions, such as complex parapneumonic effusions, empyema, or malignant effusions (14). Hepatic hydrothorax is typically thought of as a bland, transudative fluid, resulting from the changes in the hydrostatic and oncotic pressure gradients that occur commonly with portal hypertension and cirrhosis (21–23). However, trapped lung has been described as a rare complication in patients with ESLD and hepatic hydrothorax as well (15–17). Our data reveal that pleural effusions among the TL cohort were nearly all exudative by Light’s criteria and one quarter were due to empyema. Also, although some of our data is limited, there is a suggestion that there was a notable prevalence of associated pneumonia and sepsis further supporting the role of inflammation in the pathophysiology and development of pleural effusions and TL after OLT.

Additionally, those with TL more often had pleural effusions and pleural interventions prior to transplant, suggesting a preexisting complex pleural space even before transplant. In fact, one fifth of patients also had evidence of TL prior to transplant. We suspect that ongoing repeated pleural interventions can introduce trauma and consequent pleural inflammation, allowing for formation of a thick pleural rind. The consistent presence of the pleural effusion then keeps the lung in the atelectatic position, with the resultant fibrous rind restricting lung expansion on subsequent drainage procedures.

Taken together, this suggests that there are two possible contributors to poor outcomes in postoperative TL. At the local level, mechanical and functional impairment of the pleura, as evidenced by the presence of an exudative pleural effusion, leads to an increased risk of development of trapped lung. The TL itself or the presence of pleural infection results in the need for additional surgical interventions, which is associated with significant risk in this patient population (18). Additionally, TL likely also represents the consequence of systemic inflammatory pathophysiologic processes that lead to exudative effusion, empyema, and systemic illness, all of which put the patient at added risk for poor outcomes.

The optimal management of trapped lung, when found, is not clear. However, taken together with the known pathophysiologic origins of TL, our results allow us to propose some potential strategies for management. First, thorough investigation for underlying reversible systemic infection or inflammatory process should be undertaken. Our study was not designed for nor large enough to comment on implications for the liver transplant candidacy selection process. Within these limitations, we posit that it is possible that optimization of chronic conditions such as diabetes and renal failure pre-transplant serve as modifiable risk factors for TL. Additionally, those with trapped lung are much more likely to have multiple pleural procedures and require thoracic surgical intervention, which is associated with significant morbidity and mortality in the postoperative period after OLT (18). In the population of patients with ESLD, asymptomatic patients with pneumothorax ex-vacuo who have suspected trapped lung may benefit from observation and conservative management, and limited surgical interventions only when absolutely necessary (17). In fact, small studies have suggested that some cases of TL may spontaneously resolve on their own (17). This prior work and our data demonstrate that avoiding procedural pleural space intervention may be the most appropriate approach in post-OLT patients with TL or those at high risk for developing TL. Concurrently, when diagnostic thoracentesis is performed, pleural manometry and evaluation of pleural elastance may help confirm the diagnosis of TL and clarify the risk of additional procedures (24).

Our study is limited by its retrospective nature that limits full causal understanding of our findings. The size of our cohort was not adequate to determine if procedural intervention was a necessary treatment of the trapped lung, or rather if the pleural interventions increased the risk of development of TL or the risk of poor outcomes after TL had formed. Although several important preclinical factors were chosen and IPTW was performed, many other unmeasured preclinical factors could have affected outcomes and were not measured or were incomplete. Some clinical characteristics were unbalanced in the original cohort and were not included in the model, and thus could have contributed to residual selection bias. Due to a low event rate and high correlation with trapped lung, pre-operative chest tube placement was a potential confounder. Further study would be required to disentangle the effect of pre-OLT chest tube placement from trapped lung. The retrospective nature of our cohort also limited the depth of the data we otherwise would have wanted to collect. We would have liked to follow all patients with persistent pleural effusion with serial imaging post-operatively. Similarly, our study would have benefited from additional pre-operative pleural fluid analysis and infectious studies. However, since we are a quaternary referral center, many of the patients were referred from outside facilities and systemic assessment of pre-operative characteristics was not available, thus limiting thorough determination of pre-OLT risk factors. Lastly, our data represent the findings of a single high volume, high acuity liver transplant center. TL may not be seen, at least in this frequency, at other institutions who transplant at lower MELD scores or transplant patients with fewer comorbidities.

Several strengths of our analysis, however, merit emphasis. This is, to our knowledge, the largest cohort of OLT recipients with persistent pleural effusions and diagnosed trapped TL. Despite the retrospective nature of the study, all patients were longitudinally followed over at least 2 years with well-defined pre- and postoperative imaging, clinical characteristics, and adequately reported outcomes. While previous data suggests that atelectasis is a common complication after OLT, focus on TL lung as a chronic and more clinically significant form of atelectasis addresses an underreported problem and one of the most frequent reasons for thoracic surgery interventions.

## Conclusion

In patients with clinically significant, persistent pleural effusions after OLT, trapped lung is a frequent complication and is associated with increased morbidity, mortality, and health care utilization in the post-OLT period. Awareness of the risk factors for postoperative trapped lung, such as preoperative trapped lung, may be helpful with evaluation and determination of transplant risk stratification. Moving forward, a targeted evaluation of persistent pleural effusions in patients who have undergone OLT should be performed to further characterize these patients and their postoperative clinical outcomes. A better understanding would inform strategies to reduce the incidence of trapped lung, assess risk for transplant candidacy, and optimize the management of this common complication after orthotopic liver transplantation.

## Data Availability

The raw data supporting the conclusion of this article will be made available by the authors, without undue reservation.

## Abbreviations

ESLD, end-stage liver disease; HR, hazard ratio; IPTW, inverse probability of treatment weighting; IRR, incidence rate ratio; LDH, lactate dehydrogenase; MELD, Model for End-Stage Liver Disease; OLT, orthotopic liver transplantation; OR, odds ratio; TL, trapped lung; VATS, video-assisted thoracoscopic surgery.
