# Supplementary material for: Postoperative Trapped Lung After Orthotopic Liver Transplantation is a Predictor of Increased Mortality
Source: Transpl Int. 2022 May 3;35:10387. doi: 10.3389/ti.2022.10387 (PMC9110663; doi:10.3389/ti.2022.10387)
Supplement: Supplementary file 1 [file Table1.docx]

**Table s1.** Baseline characteristics of the study cohort.

Characteristics are compared by subgroup (trapped lung vs. no trapped lung) and shown before and after inverse probability of treatment weighting. Covariates chosen to create the propensity score model are shown in bold. Variables which could not be included in the model due to data constraints are indicated with superscripts. p-values reaching significance are also bolded.

HBV, hepatitis B virus; HCC, hepatocellular carcinoma; HCV, hepatitis C virus; HD, hemodialysis; intraop, intraoperative; MELD, Model for End-Stage Liver Disease; OLT, Orthotopic Liver Transplantation; thora = thoracentesis; vent = ventilator.

^a^More than 10 observations missing.

^b^Less than 5% event rate observed.
