# Supplementary material for: Postoperative Trapped Lung After Orthotopic Liver Transplantation is a Predictor of Increased Mortality
Source: Transpl Int. 2022 May 3;35:10387. doi: 10.3389/ti.2022.10387 (PMC9110663; doi:10.3389/ti.2022.10387)
Supplement: Supplementary file 2 [file Table2.docx]

**Table s2.** Clinical outcomes of the study cohort.

Clinical outcomes of the study cohort stratified by the presence of trapped lung after orthotopic liver transplantation and shown after inverse probability of treatment weighting. p-values reaching significance are bolded.

HR, hazard ratio; IRR, incident rate ratio; OR, odds ratio.
